# Effects of Dietary Fish Meal Replacement with Composite Mixture of Chicken Meal, Krill Meal, and Plant Proteins on Growth, Physiological Metabolism, and Intestinal Microbiota of Chinese Perch (*Siniperca chuatsi*)

**DOI:** 10.1155/2023/2915916

**Published:** 2023-12-27

**Authors:** Liyun Ding, Jiacheng Chen, Yanping Zhang, Jun Xiao, Xiandong Xu, Haixing Zhang, Qingtang Chen, Yuxiang Zhao, Wenjing Chen

**Affiliations:** ^1^Poyang Lake Fisheries Research Centre of Jiangxi Province, Jiangxi Fisheries Research Institute, Nanchang 330039, China; ^2^Fujian Tianma Science and Technology Group Co., Ltd., Fuqing 350300, China; ^3^State Key Laboratory of Freshwater Ecology and Biotechnology, Institute of Hydrobiology, Chinese Academy of Sciences, Wuhan 430072, China; ^4^Zhejiang University, Hangzhou 310058, China

## Abstract

This trial aimed to investigate the influence of graded replacing fish meal (D1: 0.00%, D2: 27.27%, and D3: 54.55%) with mixed protein ingredients (i.e., chicken meal, krill meal, fermented soybean meal, and soy protein concentrate) on the growth performance, muscle nutritional composition, blood biochemical indices, gut bacterial community, and transcriptome of Chinese perch. Two hundred seventy Chinese perch were divided into three groups (90 per group) and the diet lasted for 56 days. Results showed that the weight gain rate and specific growth rate were significantly lower, and the feed conversion ratio was significantly higher in the D3 group than in fish fed D1 (*P* < 0.05), with no significant differences between the D1 and D2 groups (*P* > 0.05). The muscle crude protein content was highest in the D2 group, and the crude fat content was significantly different in the order: D3 > D1 > D2 (*P* < 0.05). The levels of serum triglycerides (TG) and low-density lipoprotein cholesterol in the D2 group were significantly lower than those in the D1 group (*P* < 0.05), but there was no significant difference compared to the D3 group (*P* > 0.05). The microbial community structure changed significantly. *Mycoplasma* showed the highest abundance in the D1 and D2 groups (*P* < 0.05), and *Cetobacterium* peaked in D2 group, and significantly higher than that in D1 group (*P* < 0.05). Network analysis and cohesion index calculation showed that both network complexity and cohesion peaked in D2 group, and *Cetobacterium* was highly correlated with the cohesion index (*P* < 0.05). Further, muscle transcriptome analysis results showed that compared with the control group, differentially expressed genes were clustered (*Q* < 0.05) in the arginine and proline metabolism pathways in D2 group. Fish in D3 group significantly (*Q* < 0.05) affected genes involved in KEGG pathways of ribosome, circadian rhythm, thermogenesis, insulin signaling pathway, fatty acid degradation, oxidative phosphorylation, and apoptosis. In conclusion, under the experimental conditions, the replacement of 27.27% of fish meal by the compound protein did not have a negative impact on the growth performance of Chinese perch and could improve muscle quality, lipid metabolism, and the interaction of intestinal microbiota.

## 1. Introduction

Fish meal is widely used as a high-quality protein source in the aquatic feed production, due to the advantages of well-balanced amino acids and high palatability. However, as a result of the decline in wild fisheries resources due to factors such as overfishing and global warming, global fish meal production cannot meet the needs of the rapid development of aquaculture [1, 2]. Therefore, the search for protein sources to replace fish meal in aquafeed has become one of the highly researched topics in aquatic animal nutrition [3, 4]. At present, alternative sources of fish meal for aquafeed mainly include animal protein [5, 6], plant protein [7, 8], and insect protein sources that have emerged in recent years [9, 10]. Studies have shown that several animal and plant proteins could effectively replace fish meal in aquatic feed. Chicken meal has a wide range of sources and high-protein content, which was used as a substitute for various carnivorous fish. Antarctic krill meal contains nucleotides, glutamic acid, proline, glycine, and other rich food substances [11] and can partially or completely replace fish meal in the diet of Pacific white shrimp (*Litopenaeus vannamei*) [5], large yellow croaker (*Larimichthys crocea*) [12], European sea bass (*Dicentrarchus labrax*) [13], etc. As high-quality plant protein sources, fermented soybean meal and soy protein concentrate play an important role in cost saving for carnivorous fish feed. However, the sole plant or animal protein source has the disadvantages of amino acid composition imbalance and antinutritional factors, which always presented growth suppression, low feed utilization, and metabolic disorder after dietary fish meal replaced at a higher ratio [14, 15]. In other species, the use of a mixture of animal and plant protein sources as a substitute has proved to be a good option because of the advantages of nutritional complementarity, more balanced amino acid profiles, and lower cost [16, 17]. So, the use of a mixture of animal and plant protein sources to replace fish meal can improve the substitution effect and reduce the overdependence on fish meal in Chinese perch.

Chinese perch (*Siniperca chuatsi*) belongs to the order *Perciformes* and the genus Mandarin. In 2012, China's *Siniperca chuatsi* aquaculture output was 281,500 tons, which increased to 374,000 tons in 2021 [18], with an annual output value of more than 20-billion yuan, and has become one of the important species of freshwater aquaculture in China. However, at present, Chinese perch farming is mainly based on the combination of chilled fish and live bait [19], which not only pollutes the water environment but also makes it prone to disease outbreaks [20]. Therefore, the use of artificial diet could be helpful in the sustainable development of the Chinese perch industry. Research has shown that Chinese perch can be domesticated and fed on artificial formula feed through some special methods [21]. However, as a typical carnivorous fish, its feed contains over 55% fish meal [22]. In view of this, this study ascertained the suitability of a mixture of animal and plant protein sources (chicken meal, krill meal, fermented soybean meal, and soybean protein concentrate) to replace fish meal in the diet of Chinese perch. The study explored the effects of fish meal substitution levels on the growth, physiological metabolism, and intestinal health of Chinese perch feed, in order to provide a theoretical basis and practical reference for the formulation of Chinese perch feed and the rational use of compound protein sources in its commercial feed.

## 2. Materials and Methods

### 2.1. Test Fish

The Chinese perch was purchased from Poyang Le'an Special Aquaculture Co., Ltd., and was acclimated for 1 month before the main trial, using commercial feed in the early stages, and control feed in the later stages [21].

### 2.2. Test Feed

The basal (control) feed was formulated with fish meal as the main protein source and fish oil as the main lipid source. Based on the previous reports [22], feed with above 55% fish meal was designated as the suitable diet for Chinese perch. The control group contained 55% fish meal, and the two test groups gradually reduced the content of fish meal by 15% to 40% and 25%, with chicken meal, krill meal, fermented soybean meal, and soybean protein concentrate were used to formulate a compound protein ingredient, replacing 0.00%, 27.27%, and 54.55% of fish meal, respectively. Crystalline methionine and lysine were supplemented to reach the same level as the control group. All raw materials after crushing and sieving through an 80-size meshes were mixed proportionally according to their dietary groups, and named D1 (0.00% replacement), D2 (27.27%), and D3 (54.55%), respectively. The mixed raw materials were formed into pellets with 2-mm diameter and 4–5-mm length by the feed machine. After air drying at room temperature, the diets were placed in a −20°C refrigerator and stored tightly for later use. The nutrients and amino acid composition of the experimental feed are shown in Tables 1 and 2.

### 2.3. Test Set Up and Management

The trial was conducted in pond cages with dimensions of 1.0 m × 1.0 m × 2 m with net cover. At the beginning of the experiment, the domesticated Chinese perch were starved for 24 hr, the physically robust evenly sized fish were distributed into nine groups and stocked in cages. Each group had 30 fish with an initial weight of 46.33 ± 2.08 g and the groups were randomly assigned to the test feeds in triplicate. The feeding period lasted for 56 days, with the fish fed to apparent satiation twice a day (6:00 and 18:00). Water quality was tested regularly: temperature ranged 26–32°C, ammonia nitrogen was less than 0.1 mg/L, and the pH ranged 7.5–8.5. The water was continuously aerated to keep the dissolved oxygen at 7.0–9.3 mg/L.

### 2.4. Sample Collection and Analyses

After the breeding experiment, the fish were starved for 24 hr and then anesthetized with MS222 for weighing and counting. Twelve fish were randomly taken from each tank to measure individual body length and weight. This was followed by blood collection from the tail vein, and the fish was dissected for liver, intestines, and muscle samples. Intestines from six fish were put in sterilized EP tubes and stored at −80°C for the detection of intestinal microbiota.

Proximate compositions of feed and muscle were determined with methods of AOAC [23]. The moisture content was determined by drying in an oven at 105°C to constant weight. The crude protein, crude fat, and crude ash contents were determined by Kjeldahl nitrogen method, Soxhlet extraction method, and 550°C burning, respectively.

The levels of triglyceride (TG), total cholesterol (TC), high-density lipoprotein cholesterol (HDLC), low-density lipoprotein cholesterol (LDLC), and glucose (GLU) in pooled serum were determined by an automatic Chemistry Analyzer (Hitachi 7600, Tokyo, Japan).

### 2.5. DNA Extraction and High-Throughput Sequencing

According to the product instructions, each sample was extracted with the PowerFecal® DNA Isolation Kit. The V3-V4 region for 16S rRNA gene was amplified by 338F/806R and sequenced by Illumina MiSeq (MAGIGENE Biological Technology Co., Ltd., Guangzhou, China) [24].

### 2.6. Molecular Ecology Networks

The microbial network was constructed as previously described by Zhao et al. [25]. In detail, Spearman correlation of log-transformed OTU numbers was calculated and RMT module was used to screen the optimal cutoff threshold [26, 27]. The topology indices of the constructed network and random networks were calculated with MENAP interface. Gephi 0.9.2 was used to visualize the constructed networks.

### 2.7. Cohesion Calculation

To quantify the interaction index between various microbes, cohesion was calculated according to Equation (1) [28]. In detail, all positive correlations were averaged based on the total number of samples to obtain a connection matrix. From the insight of each sample, positive and negative cohesions were the sum of abundance-weighted, null positive and negative correlations were according to the model calibration. Thus, the degree of collaborative or competitive interactions could be calculated as follows:

(1) $$\begin{array}{c} \text{Cohesion index }=\sum_{i=1}^{m}{\text{abundance}}_{i}\times {\text{connectedness}}_{i}. \end{array}$$

### 2.8. Muscle Transcriptome Analysis

The total RNA of all samples was extracted using Trizol reagent (Invitrogen, Waltham, MA, USA), the quantity and quality of extracted RNA were detected by ND 2000 (NanoDrop Technologies) and 2100 Bioanalyzer (Agilent), respectively. Genomic DNA remained was removed with DNase I (TaKara), then the isolated mRNA was reverse transcribed into cDNA with added adapters. The obtained cDNA libraries were constructed and sequenced by WebShenzhen Huada Gene Technology Co., Ltd., with the Illumina HiSeq PE 2X150bp read length.

The raw paired end reads were trimmed and quality controlled to obtain clean data. Later, clean data were de novo assembled using Trinity and searched to identify proteins to retrieve their function annotations. Transcript expression value was calculated using the fragments per kilobase of exon per million mapped reads (FPKM) method, while the software package RSEM was used to quantify gene and isoform abundances.

### 2.9. Validated RNA-Seq Results Using qPCR

PrimeScript RT Reagent Kit (Takara, Japan) was used to reverse-transcribe 1-*μ*g RNA into cDNA following the manufacturer's instructions, and eight related genes were selected for qPCR validation. The qPCR was performed with Bio-Rad CFX-96 real-time PCR system, using a total volume of 20 *μ*L containing SYBR Green Real-time PCR Master Mix (10 *μ*L), primers (0.2 *μ*L and 10 *μ*m), diluted cDNA template (1 *μ*L), and ultrapure water (8.6 *μ*L). The PCR program involved 95°C for 30 s, 40 cycles of 95°C for 5 s, 60°C for 30 s. The comparative Ct method was used to calculate the relative expression levels of target genes with *β*-actin as the reference gene. All reactions were performed in triplicate, and the results were presented as mean ± S.D.

### 2.10. Data Processing and Analysis

Weight gain rate (WGR, %) = 100 × (final weight−initial weight)/initial weight

Specific growth rate (SGR, %/d) = 100 × (Ln final weightLn initial weight)/test day

Feed conversion ratio (FCR) = dry feed consumed/(final weightinitial weight)

Feed intake (FI, g/100 g/days) = dry feed consumed/[(final fish weight + initial fish weight)/2]/feeding days

Survival rate (SR, %) = final fish number/initial fish number × 100

Condition factor (CF, g/cm^3^) = 100 × weight/body length^3^

Hepatosomatic index (HSI) = 100 × liver weight/body weight

Viscerosomatic index (VSI) = 100 × visceral weight/weight.

One-way ANOVA was performed using SPSS 25.0 software, and the difference was compared for significance by Tukey's multiple range test, with the significance level set at *P* < 0.05. The results are presented as mean ± SE.

## 3. Results

### 3.1. Growth Performance

As shown in Table 3, there were no significant differences in HSI, VSI, CF, FI, and SR between the experimental groups (*P* > 0.05). There was no significant difference in WGR and SGR between the D2 and D1 groups (*P* > 0.05), but both were significantly higher than fish fed D3 (*P* < 0.05). The FCR of the D3 fish was significantly higher than that of fish fed both D1 and D2 (*P* < 0.05), and there was no significant difference in the FCR of fish from D1 and D2 groups (*P* > 0.05).

### 3.2. Muscle Nutritional Composition

According to the results in Table 4, replacing fish meal with the compound protein significantly affected the nutritional indexes of Chinese perch muscle. The muscle crude protein content of fish fed D2 was significantly higher than that of the D1 group (*P* < 0.05). However, there was no significant difference between the D2 and D3 groups (*P* > 0.05). The muscle crude lipid content in the D2 group was significantly lower than that of the other groups (*P* < 0.05).

It can be seen from Table 5 that replacement of fish meal by the compound protein significantly affected the amino acid content of Chinese perch muscle. The muscle Val, Phe, Leu, Ile, Thr, *∑*EAA, Asp, and Glu contents of the D2 group were significantly higher than those of the D1 and D3 fish (*P* < 0.05). The total AA content (*∑*AA) in the muscle of fish from the D2 group was significantly higher than that of the D3 group (*P* < 0.05), but not significantly higher than that of the D1 group (*P* > 0.05). There was no significant difference in *∑*NEAA between groups (*P* > 0.05).

### 3.3. Blood Biochemical Indices

The replacement of fish meal by the compound protein significantly affected the blood lipid biochemical indices of Chinese perch (Table 6). The levels of serum triglycerides (TG) and LDLC in the D2 group were significantly lower than those in the D1 group (*P* < 0.05), but there was no significant difference compared to the D3 group (*P* > 0.05). There were no significant differences in serum TC, HDLC, and GLU levels between groups (*P* > 0.05).

### 3.4. The Succession of Microbial Community

**Figure 1:**
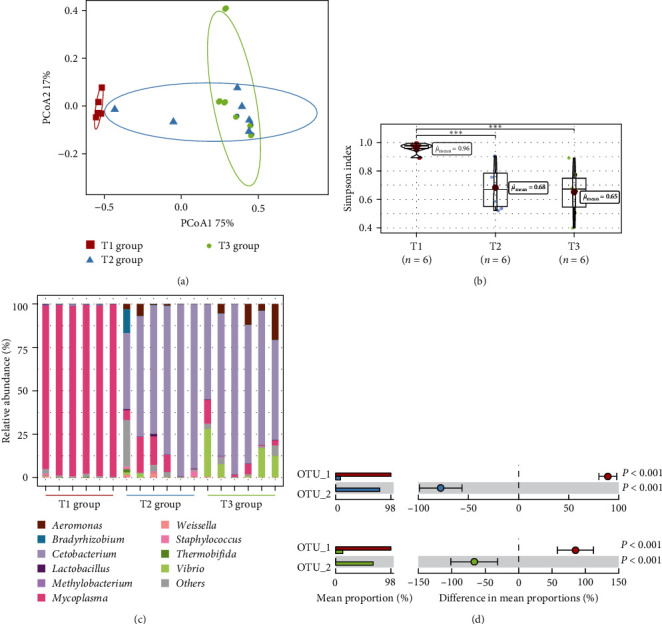
Succession of intestinal microbial community: (a) PCoA analysis, (b) Simpson index, (c) Relative abundance in genera level, and (d) STAMP analysis.

High-throughput sequencing was used to reveal the succession of microbial communities under the dietary modifications (Figure 1). Results of principle coordinates analysis (PCoA) showed a differential community clustering between nonsubstituted (D1) group and substituted (D2 and D3) groups (Figure 1(a)). Moreover, the Simpson index peaked in the D1 group and significantly higher than D2 and D3 groups (*P* < 0.05) (Figure 1(b)). The relative abundance at the genus level showed that *Mycoplasma* was the most abundant genus in D1 group, accounting for 97.6%. Meanwhile, the abundance of *Mycoplasma* decreased to less than 0.1% in the substituted groups, with *Cetobacterium* dominating the microbial community (76.7%) (Figure 1(c)). STAMP analysis was further employed to determine significant differences in microbes among the three groups (Figure 1(d)). It showed that the OTU_1 and OTU_2 were significantly different between the D1 (T1) and D2 (T2) groups and between the T1 and D3 (T3) groups. As expected, OTU_1 was affiliated with *Mycoplasma* and OTU_2 affiliated with *Cetobacterium*. In contrast, small differences in the Simpson index and nonsignificantly different genera between occurred between D2 and D3 groups.

### 3.5. Molecular Network Construction and Cohesion

**Figure 2:**
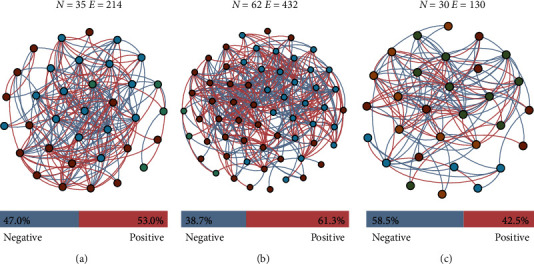
Molecular network construction: (a) D1 group, (b) D2 group, and (c) D3 group.

To reveal the potential microbial interaction, microbial networks were constructed based on the dietary modification (Figure 2). The network structures were significantly altered between treatments due to dietary modification. The average degree (avg*K*) in D2 (avg*K* = 13.9) and D3 group (avg*K* = 12.2; i.e., potential microbial interaction) was 1.4–1.6 times higher than that in D1 group (avg*K* = 8.2; Table S1). However, an opposite trend was observed in the total number of nodes and edges. The maximum value of total nodes and edges was observed in the D2 group (*N* = 62, *E* = 432), and the minimum value was observed in the D3 group (*N* = 30, *E* = 130).

**Figure 3:**
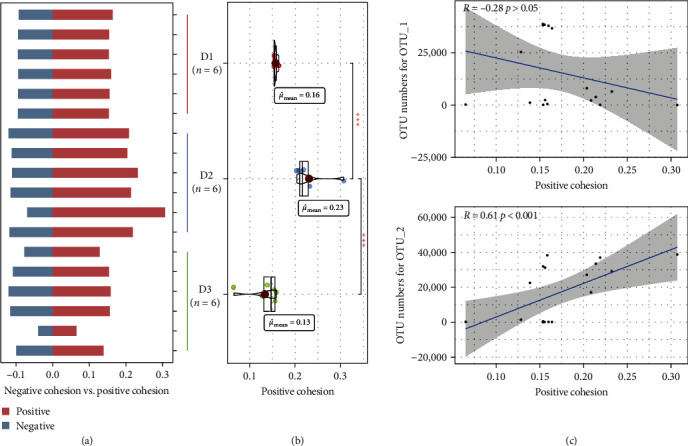
Cohesion index in gut microbes: (a) calculation of cohesion index, (b) positive cohesion in carious group, and (c) correlation of OTU_1 and OTU_2 with positive cohesion.

To confirm the changes of microbial interaction under different dietary modifications, the cohesion index was further calculated (Figure 3). The possible cooperation and competition behavior among the groups are shown in Figure 3(a). According to the improvement of positive links in the network analysis, the positive cohesion was focused on (Figure 3(b)). Remarkably, the positive cohesion was significantly improved in the D2 group, which was 1.4–1.8 times higher than that in the D1 and D3 groups. Due to the significant differences between OTU_1 and OTU_2 in the different treatments, we further compared their correlation with positive cohesion. It showed that the OTU numbers of OTU_1 was negatively associated with the positive cohesion, and that of OTU_2 was positively associated with the positive cohesion (Figure 3(c)).

### 3.6. Summary of Muscle RNA Sequencing and Identification of Differentially Expressed Genes (DEGs)

In this study, Q30 value is larger than 90.63% and Q20 value is larger than 96.05% which indicates that sequencing accuracy for each replicate had reached 99.9% and met the requirement for further analysis (Table S3). As it is depicted in Table S4, 30 genes were upregulated and 17 genes were downregulated in D2 group, 1,379 genes were upregulated, and 672 genes were downregulated in D3 group when compared with the control group.

### 3.7. KEGG Enrichment Analysis

As Table 7 shows, the DEGs of arginine and proline metabolism pathways clustered in D2 group (*Q* < 0.05).Genes involved in KEGG pathways of ribosome, circadian rhythm, thermogenesis, insulin signaling pathway, fatty acid degradation, oxidative phosphorylation, and apoptosis are affected significantly in D3 group (*Q* < 0.05).

### 3.8. Validating Related DEGs

**Figure 4:**
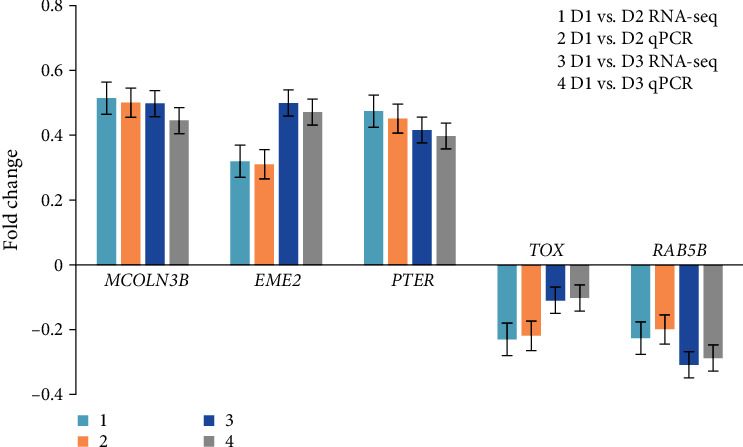
Detecting differentially expressed genes with qPCR. MCOLN3B, mucolipin TRP cation channel 3b; EME2, essential meiotic structure-specific endonuclease subunit 2; PTER, phosphotriesterase related; TOX, thymocyte selection associated high-mobility group box; RAB5B, member RAS oncogene family.

Five related DEGs, including MCOLN3B, EME2, PTER, TOX, and RAB5B, were selected for the validation using quantitative real-time PCR (qRT-PCR). The fold changes obtained from qRT-PCR were compared to those obtained from the differential gene expression (DGE) analysis. The results showed a correlation between the trends of qPCR and RNA-seq data, indicating the accuracy of the deep-sequencing data (Figure 4). The primers for the different genes were shown in Table 8.

## 4. Discussion

As a typical ferocious, carnivorous fish, more than 55% of high-quality fish meal is often added to the diet of Chinese perch [22]. This makes their feed expensive and affects profitability. In this study, replacing 27.27% fish meal with animal and plant complex proteins in the diets for Chinese perch had no adverse effect on the WGR and SGR. Remarkably, the WGR and SGR of Chinese perch would significantly decreased when the substitution level reached 54.55%. With the further improvement of substitution level, the FCR also significantly increased, and the low level of feed utilization limited the growth of Chinese perch. Indeed, a high proportion of fish meal substitution would inhibit the growth of Chinese perch, as described previously [29], which might be caused by the antinutrients or imbalance of amino acid ratio in the feed. Since carnivorous fish have a relatively high demand for dietary protein, excessive use of plant proteins with an amino acid imbalance (lacking lysine and methionine) as a substitute for fish meal can result in a series of negative effects, such as growth inhibition, decreased immune function, and deterioration of meat quality [30, 31]. To reduce the negative impact of excessive replacement of fish meal with plant protein, more and more studies are tending to use more balanced composite animal and plant protein sources to replace fish meal in diets. In general, compared with the replacement of a single plant or animal protein source, mixed protein sources replace fish meal better, as the nutritional components complement each other, which can not only increase the proportion of alternative fish meal, but also be more conducive to improve the nutrient absorption and utilization, and health of farmed animals [16, 32]. Wang et al. [33] showed that compound animal and plant protein composed of fly maggot meal, peanut meal, corn gluten meal, soybean meal, and gluten meal successfully replaced fish meal, and under the same substitution ratio, had a higher WGR than the compound plant protein group. Similarly, replacing fish meal with a mixed animal and plant protein sources consisting of shrimp hydrolysate and plant protein mixture improved the growth performance of largemouth bass, and the use of compound protein reduced the fish meal content in their feed to 30% [16].

The TG and TC in serum are mainly from the liver, and their changes reflect the body's absorption and metabolism of lipids. HDLC and LDLC reflect the catabolism and transport of lipids in the body [34, 35]. In this study, the substitution of 27.27% and 54.55% fish meal with composite protein sources in diets significantly reduced the levels of TG and LDLC in the serum of Chinese perch, indicating that compound protein enhanced the body's absorption and utilization of lipids, improved the body's blood lipid levels and lipid metabolism function. This may be because the compound protein in this experiment contains krill meal, fermented soybean meal, and soy protein concentrate. Studies have shown that krill meal can regulate the dynamic balance of animal lipid metabolism, as krill meal is rich in n-3 unsaturated fatty acids and phospholipids, which can lower the levels of TG and TC in blood and stimulate mitochondria to regulate the fat metabolism [36]. Fermented soybean meal and soybean protein concentrate contain soy isoflavones and plant sterols, which have the effect of reducing blood lipids and clearing cholesterol [37, 38]. Pham et al. [39] have also indicated that partially replacing fish meal with complex proteins significantly reduces the serum TG levels of *Trachinotus blochii* and influences lipid metabolism.

The biochemical composition of aquatic animals is closely related to the nutritional content of their food [29], and the nutrient content of fish muscle as the main edible part of the fish is an important quality indicator [40]. The replacement of fish meal with a complex protein preparation resulted in a significant increase in crude protein content and a decrease in crude fat content in the present study. These results highlighted that an appropriate proportion of compound protein sources replacing fish meal could increase the deposition efficiency of protein in the feed of Chinese perch. It might be caused by the animal and plant protein sources used in this experiment can supplement each other with nutrients and other factors, causing a synergistic effect and promoting the digestion and absorption of protein. As the basic unit of protein, changes in the composition and content of amino acids affect the nutritional value of muscle [41]. The results of this trial showed that the most abundant amino acid in mandarin muscle was Glu, followed by Asp, Lys, and Leu, consistent with the results of carnivorous fish such as grouper and largemouth bass [42, 43]. Flavor is the complex sensation of touch, taste and smell in the mouth, and the content of essential amino acids, umami amino acids, and aromatic amino acids in muscle is closely related to flavor [44]. In this experiment, when the compound protein preparation replaced 27.27% fish meal, the content of the essential amino acids His, Val, Phe, Leu, Ile, Thr, and *∑*EAA, the total umami amino acids, Asp and Glu increased significantly. The significant increase means that the nutritional value and flavor of Chinese perch muscles can be significantly improved by replacing fish meal with an appropriate proportion of compound protein sources. With a further increase in the proportion of compound protein substitution, Chinese perch showed a downward trend in muscle crude protein and amino acid content, which is similar to the results in humpback bass (*Cromileptes altivelis*) [45], and Pacific white shrimp [46], and may be due to the lower digestibility of protein and amino acids in a diet with high proportion of plant protein raw materials. Therefore, a high proportion of fish meal substitution can reduce the utilization of protein and amino acids, thereby affecting the deposition rate of proteins and amino acids in the body [47]. Further transcriptome analysis demonstrated the muscle function change after different proportion of fish meal substitution. Based on the transcriptome data, fish in D2 group had relatively low influence compared with the D3 group. Fish in D3 group significantly affected genes involved in KEGG pathways of insulin signaling pathway, fatty acid degradation, and oxidative phosphorylation, which were highly related to energy metabolism. Oxidative phosphorylation is the process by which ATP synthesis (the most efficient ATP synthesis way) is coupled to the movement of electrons through the mitochondrial electron transport chain and the associated consumption of oxygen [48]. The insulin signaling pathway is a highly conserved and critical regulator of metabolism in mammals and in flies, where it senses organismal nutrient levels to regulate multiple physiological functions including carbohydrate metabolism and lipid metabolism [49]. Therefore, the above changed pathways might be associated with increased muscle crude lipid content after a high proportion of fish meal substitution in D3 group.

Gut microbes also play an essential role in regulating the immune response, nutrition, and metabolic homeostasis [50]. In contrast to rainbow trout, which is also a carnivorous fish, *Mycoplasma* was the predominant genus in the gut microbes of *S. chuatsi* under fish meal-rich diets [51]. The large proportion of vitamins and amino acids contained in conventional diets could provide a suitable environment for its dominance since it cannot synthesize them. As expected, replacement of fish meal with the compound protein preparation directly led to changes in microbial communities and diversity. The selective stress created by changes in diet modification would drive the targeted succession of the microbial community [52]. Significant differences in Simpson index (the lower the Simpson's index the higher the diversity) could be observed between the gut microbes of fish fed the fish meal-rich diet (D1 group) and the compound protein preparation diet (D2 and D3 groups). The increase in diversity among the substituted groups can be attributed to higher level of plant-based and other animal protein ingredients, due to the possibility of increased stress from plant-based and other animal protein ingredients promoting metabolic diversity [53]. As expected, OTU_1_*Mycoplasma* and OTU_2_*Cetobacterium* showed significant differences with diet modification, while no significant differences were observed between D2 and D3 groups. Thus, the addition of compound protein preparation could have selectively enriched genus *Cetobacterium*, which has the capacity to regulate glucose homeostasis in fish [54]. Due to the weak carbohydrate utilization by carnivorous species in fish, the high carbohydrate levels in the fish diet (mainly starch) might lead to persistent postprandial hyperglycemia [55]. The enhancement of *Cetobacterium* could be a promising avenue to improve glucose homeostasis [54]. Although the abundance of *Cetobacterium* improved with the compound protein preparation diet, nearly all the indicators of fish quality were better in D2 group than in the D3 fish. Thus, fish could regulate the relative abundance of *Cetobacterium* to adapt to changes in diet, but excessive substitution with compound protein preparation might be harmful to fish quality.

The microbial network was further assessed to reveal how diet modification influenced the relationship between gut microbes. The substitution of composite protein preparations looked to have influenced the network complexity, which indicates potential microbial interaction. The value of avg*K*, total nodes, total edges, and cohesion index also confirmed the alteration of potential interactions with substitution of composite protein preparations. Although the relative abundance of *Cetobacterium* was similar in D2 and D3 groups, a significant difference in network status was observed. Compared to D1 group, the microbial interactions were significantly strengthened in D2 group, but significantly reduced in D3 group. In D2 group, *Cetobacterium* was highly associated with 19 nodes, whereas groups D1 and D3 had only 3 (Figure S1). Thus, *Cetobacterium* might act as an ecological engineer to strengthen microbial cooperation and stimulate function [56]. The nodes positively associated with *Cetobacterium* in D2 group were affiliated with *Lactobacillus*, *Bacillus*, *Turicibacter*, *Aeromonas*, *Shewanella*, and other genera important for immune response and metabolic homeostasis (Table S2). *Lactobacillus* and *Bacillus* are regarded as probiotics, which could regulate lipid metabolism [57]. *Turicibacter* might interact with bile acids, which could promote the absorption of vitamins and fats in the small intestine [58]. *Aeromonas* and *Shewanella* were common genera in the gut of freshwater fish, and can secrete BefA protein to maintain stable blood sugar in fish [59]. However, in D1 and D3 groups, all the nodes associated with *Cetobacterium* showed a negative correlation, suggesting a stronger competition. Although excessive substitution of composite protein preparations (D3 group, 54.55%) also increased *Cetobacterium* abundance, the microbial interaction would be reduced and *Cetobacterium* might compete with other microbes, leading to lower fish quality. Overall, appropriate substitution of composite protein preparations (D2 group, 27.27%) could simultaneously enhance *Cetobacterium* abundance and induce interactions with the other intestinal probiotics, thereby enhancing fish quality.

## 5. Conclusion

In conclusion, our results suggest that a composite mixture of chicken meal, krill meal, fermented soybean meal, and soybean protein concentrate can be used to reduce the amount of fish meal in Chinese perch diets. These results indicate that a suitable composite mixture of chicken meal, krill meal, and plant proteins has no adverse effect on growth performance of Chinese perch and could improve muscle quality, lipid metabolism, and gut microbiota interactions. Overall, the use of a composite mixture of chicken meal, krill meal, fermented soybean meal, and soybean protein concentrate reduced fish meal in bass diets to 40%. This can be considered in preparing cost-effective feed for this aquaculture species.

## Figures and Tables

**Table 1:** Formulation and proximate composition of the experimental diets (%, dry matter basis).

Ingredients (%)	Diets		
	Control (D1)	27.27% (D2)	54.55% (D3)
Fish meal	55	40	25
Chicken meal	3	7.5	12
Krill meal	3	7	11
Fermented soybean meal	3	7	11
Soy protein concentrate	3	7	11
Spirulina meal	2	2	2
Wheat gluten	3	3	3
Fish oil	6	6.3	6.6
Lysine	0	0.25	0.5
Methionine	0	0.2	0.4
Calcium dihydrogen Phosphate	1	1	1
Vitamin mix^1^	0.5	0.5	0.5
Mineral mix^2^	1	1	1
Choline chloride	0.5	0.5	0.5
Gelatinized tapioca starch	15	15	14.5
Cellulose	4	1.75	0
Total	100	100	100
Proximate composition			
Crude protein	49.3	49.8	48.9
Crude lipid	11.0	10.9	10.8
Ash	11.1	10.6	9.6
Moisture	8.9	8.5	8.7

^1^Vitamin premix (per kg of diet): vitamin A, 6,000 IU; vitamin B_1_ (thiamin), 10 mg; vitamin B_2_ (riboflavin), 10 mg; vitamin B_6_, 10 mg; vitamin B_12_, 0.05 mg; vitamin D_3_, 3,000 IU; vitamin E 60 mg; vitamin K_3_ 5 mg; vitamin C, 500; folic acid, 3 mg; biotin, 0.2 mg; pantothenic acid calcium, 40 mg; inositol, 150 mg; niacinamide, 50 mg. ^2^Mineral premix (per kg of diet): MnSO_4_, 12 mg; MgSO_4_, 12 mg; KCl, 110 mg; NaCl, 180 mg; ZnSO_4_, 25 mg; KI, 1 mg; CuSO_4_, 15.5 mg; FeSO_4_, 120 mg; Na_2_SeO_3_, 0.2 mg; Co, 2 mg.

**Table 2:** Amino acid composition of the ingredients and experimental diets (%, dry matter basis).

Amino acid contents	Ingredients					Diets		
	Fish meal	Chicken meal	Krill meal	Fermented soybean meal	Soy protein concentrate	Control (D1)	27.27% (D2)	54.55% (D3)
Essential amino acid								
Arginine	4.05	4.00	4.46	3.26	4.98	2.69	2.61	2.66
Histidine	2.15	2.22	1.49	1.42	1.81	1.43	1.40	1.35
Valine	3.60	2.45	3.69	2.35	3.40	1.98	1.87	1.95
Phenylalanine	2.40	3.21	3.68	2.50	3.40	1.98	1.94	1.95
Leucine	5.48	6.27	5.50	3.91	5.17	3.47	3.30	3.32
Isoleucine	3.93	2.46	3.58	2.29	4.23	1.53	1.42	1.65
Threonine	3.17	2.52	3.15	1.96	2.73	2.07	1.98	1.90
Methionine	2.25	1.29	2.13	0.78	0.92	1.29	1.22	1.09
Lysine	5.65	4.95	5.00	2.79	4.23	3.07	2.76	2.85
Nonessential amino acid								
Aspartic	6.29	4.11	5.63	5.77	7.76	4.54	4.37	4.15
Serine	2.50	2.22	2.60	2.50	3.50	2.51	2.54	2.54
Glutamic	9.75	6.62	6.94	9.42	12.09	7.49	7.47	7.36
Alanine	4.30	4.23	2.94	2.41	2.74	2.78	2.60	2.44
Glycine	4.30	7.06	2.55	2.13	2.74	4.26	3.83	3.75
Tyrosine	2.44	2.34	3.34	1.68	2.34	1.59	1.56	1.52
Proline	2.92	6.21	4.00	2.51	2.68	1.78	1.76	1.64
Cystine	0.61	0.61	1.28	0.76	0.98	1.18	1.12	0.97

**Table 3:** Effect of dietary fish meal replacement with protein mixtures on the growth performance in Chinese perch (*Siniperca chuatsi*).

Items	Diets		
	Control (D1)	27.27% (D2)	54.55% (D3)
Initial body weight, IBW (g)	46.33 ± 2.08	45.67 ± 3.51	46.33 ± 2.52
Final body weight, FBW (g)	102.12 ± 9.70^b^	96.64 ± 2.31^ab^	85.48 ± 5.19^a^
Percent weight gain, WGR (%)	120.14 ± 12.67^b^	112.21 ± 11.45^b^	84.46 ± 3.44^a^
Special growth rate, SGR (%)	1.41 ± 0.10^b^	1.34 ± 0.10^b^	1.09 ± 0.10^a^
Feed conversion rate, FCR	1.21 ± 0.16^a^	1.31 ± 0.03^a^	1.61 ± 0.03^b^
Feed intake, FI (g/100 g/days)	1.61 ± 0.12	1.67 ± 0.07	1.71 ± 0.08
Survival rate, SR (%)	95.56 ± 5.09	94.44 ± 1.92	93.33 ± 3.33
Hepatosomatic index, HSI (%)	1.57 ± 0.14	1.46 ± 0.14	1.38 ± 0.15
Viscerosomatic index, VSI (%)	9.16 ± 0.28	8.91 ± 0.76	8.51 ± 0.66
Condition factor, CF (%)	3.14 ± 0.29	3.01 ± 0.17	2.93 ± 0.29

*Notes*. Values in the same row with different superscripts show significant difference (*P* < 0.05).

**Table 4:** Effect of dietary fish meal replacement with protein mixtures on the muscle proximate composition in Chinese perch (*Siniperca chuatsi*) (%).

Items	Diets		
	Control (D1)	27.27% (D2)	54.55% (D3)
Crude protein	87.65 ± 0.69^a^	89.32 ± 0.51^b^	87.91 ± 0.68^ab^
Crude lipid	4.62 ± 0.41^b^	3.66 ± 0.25^a^	5.58 ± 0.34^c^

*Notes*. Values in the same row with different superscripts show significant difference (*P* < 0.05).

**Table 5:** Effect of dietary fish meal replacement with protein mixtures on the muscle amino acid in Chinese perch (*Siniperca chuatsi*) (%, dry matter basis).

Items	Diets		
	Control (D1)	27.27% (D2)	54.55% (D3)
Essential amino acid			
Arginine	5.07 ± 0.10^a^	5.15 ± 0.00^b^	5.04 ± 0.03^a^
Histidine	1.89 ± 0.02^a^	1.99 ± 0.00^b^	1.96 ± 0.03^b^
Valine	4.28 ± 0.05^a^	4.39 ± 0.02^b^	4.28 ± 0.03^a^
Phenylalanine	3.68 ± 0.03^a^	3.78 ± 0.00^b^	3.65 ± 0.04^a^
Leucine	6.82 ± 0.08^a^	6.99 ± 0.03^b^	6.79 ± 0.05^a^
Isoleucine	4.04 ± 0.05^a^	4.14 ± 0.01^b^	4.04 ± 0.04^a^
Threonine	3.93 ± 0.04^a^	4.03 ± 0.01^b^	3.88 ± 0.04^a^
Methionine	2.99 ± 0.04	2.95 ± 0.12	2.94 ± 0.08
Lysine	8.38 ± 0.13^ab^	8.55 ± 0.01^b^	8.19 ± 0.08^a^
*∑*EAA^1^	41.09 ± 0.52^a^	41.97 ± 0.14^b^	40.78 ± 0.26^a^
Nonessential amino acid			
Aspartic	8.87 ± 0.09^a^	9.09 ± 0.03^b^	8.83 ± 0.08^a^
Serine	3.46 ± 0.05	3.55 ± 0.01	3.46 ± 0.05
Glutamic	13.15 ± 0.11^a^	13.50 ± 0.12^b^	12.98 ± 0.06^a^
Alanine	5.36 ± 0.11	5.41 ± 0.00	5.31 ± 0.05
Glycine	4.36 ± 0.20	4.25 ± 0.14	4.36 ± 0.12
Tyrosine	2.81 ± 0.03^a^	2.92 ± 0.00^b^	2.81 ± 0.01^a^
Proline	2.76 ± 0.12	2.76 ± 0.07	2.85 ± 0.07
Cystine	0.85 ± 0.02	0.85 ± 0.01	0.84 ± 0.03
*∑*NEAA^2^	41.63 ± 0.69	42.33 ± 0.06	41.44 ± 0.20
*∑*AA^3^	82.72 ± 1.19^ab^	84.30 ± 0.09^b^	82.22 ± 0.38^a^

*Notes*. ^1^∑EAA, total essential amino acids; ^2^*∑*NEAA, total nonessential amino acids; ^3^∑AA, total amino acids. Values in the same row with different superscripts show significant difference (*P* < 0.05).

**Table 6:** Effect of dietary fish meal replacement with protein mixtures on the serum biochemical indices in Chinese perch (*Siniperca chuatsi*).

Items	Diets		
	Control (D1)	27.27% (D2)	51.55% (D3)
TG (m mol/L)	1.33 ± 0.08^b^	0.92 ± 0.20^a^	0.76 ± 0.12^a^
TC (m mol/L)	5.80 ± 0.57	5.28 ± 0.51	5.67 ± 0.98
HDLC (m mol/L)	2.18 ± 0.21	1.99 ± 0.24	2.19 ± 0.16
LDLC (m mol/L)	2.10 ± 0.16^b^	1.47 ± 0.23^a^	1.41 ± 0.20^a^
GLU (m mol/L)	10.74 ± 0.81	13.11 ± 2.55	10.85 ± 1.76

*Notes*. Values in the same row with different superscripts show significant difference (*P* < 0.05).

**Table 7:** KEGG enrichment among groups.

Groups	Pathway ID	Pathway name	Term candidate gene num.	*Q* value
D1 vs. D2	ko00330	Arginine and proline metabolism	3	0.01828091
	ko03010	Ribosome	103	1.62E-69
	ko04710	Circadian rhythm	19	1.51E-05
	ko04714	Thermogenesis	56	0.001647543

D1 vs. D3	ko04910	Insulin signaling pathway	38	0.01608421
	ko00071	Fatty acid degradation	16	0.03095742
	ko00190	Oxidative phosphorylation	27	0.03920796
	ko04214	Apoptosis	18	0.03920796

**Table 8:** Details of the primer sequence used for qPCR.

Primers	Sequences (5′−3′)
MCOLN3B-F	AGTTAGTCTCTTTTGGGCTG
MCOLN3B-R	TTTCTCATATTCGTGATTGC
EME2-F	TATTATCCCATCCCACCCAT
EME2-R	GCAACACAGACATTTCACAT
PTER-F	GAACACTACCACGGGCATCG
PTER-R	CCCTCAGCACCTTCGTCTCG
TOX-F	TGACTTCCTTATTGCTGCCA
TOX-R	TTTATTCCTCCTCGTGACCT
RAB5B-F	CAGCCTATTCTTGCTCGCCT
RAB5B-R	TTACGCCTGTTGGAACCTTA
*β*-Actin-F	ATCGTGCGCCCCAGGCACC
*β*-Actin-R	CTCCTTAATGTCACGCACGATTTC

*Note*. MCOLN3B, mucolipin TRP cation channel 3b; EME2, essential meiotic structure-specific endonuclease subunit 2; PTER, phosphotriesterase related; TOX, thymocyte selection associated high mobility group box; RAB5B, member RAS oncogene family.

## Data Availability

All datasets generated for this study are included in the article or supplementary material. Data will be available from the corresponding authors by reasonable request.

## Supplementary Materials

Supplementary Materials Figure S1: subnetwork for OTU_2 and its neighboring nodes. Table S1: topology parameters for constructed networks. Table S2: the OTUs linked with OTU_2 in D2 group. Table S3: summary of the RNA sequencing data. Table S4: difference analysis of expressed genes.
