# Supplementary material for: Effects of Dietary Fish Meal Replacement with Composite Mixture of Chicken Meal, Krill Meal, and Plant Proteins on Growth, Physiological Metabolism, and Intestinal Microbiota of Chinese Perch (Siniperca chuatsi)
Source: Aquac Nutr. 2023 Dec 27;2023:2915916. doi: 10.1155/2023/2915916 (PMC11221970; doi:10.1155/2023/2915916)
Supplement: Supplementary Materials — Figure S1: subnetwork for OTU_2 and its neighboring nodes. Table S1: topology parameters for constructed networks. Table S2: the OTUs linked with OTU_2 in D2 group. Table S3: summary of the RNA sequencing data. Table S4: difference analysis of expressed genes. [file 2915916.f1.docx]

Supplementary material


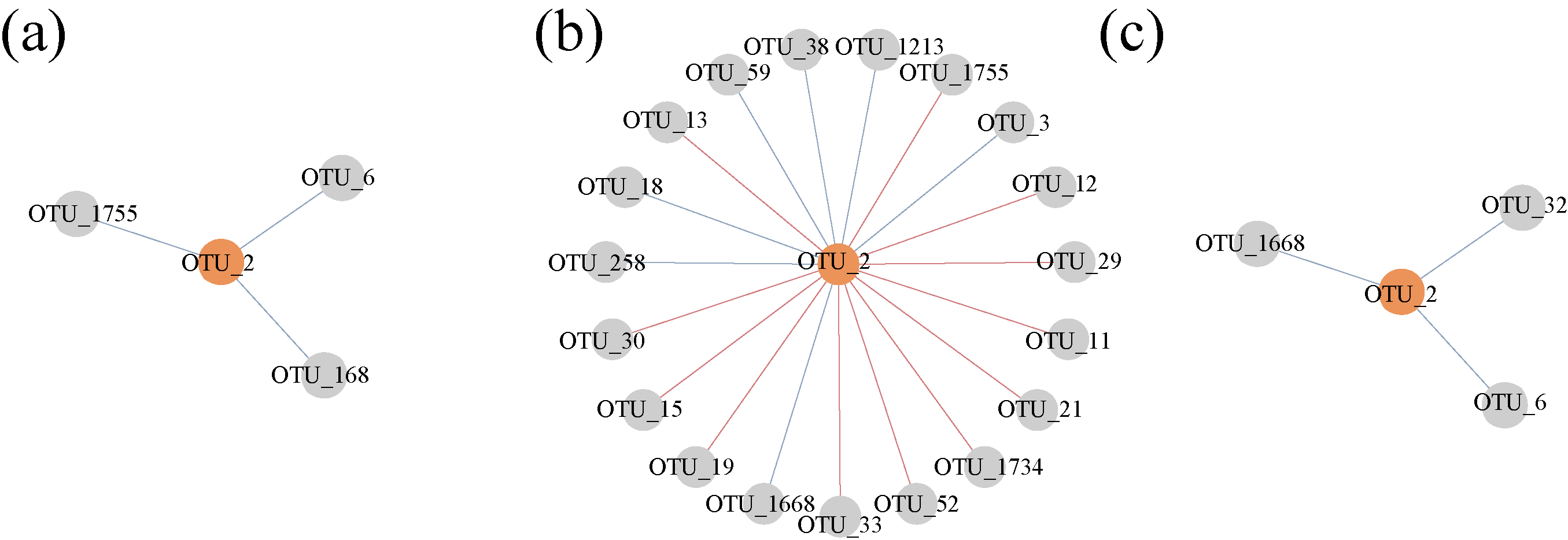


Fig S1 Sub-network for OTU_2 and its neighbouring nodes (a) D1 group. (b) D2 group. (c) D3 group.

Table S1 Topology parameters for constructed networks

|  | D1 | D2 | D3 |
| --- | --- | --- | --- |
| Total nodes | 35 | 62 | 30 |
| Total links | 214 | 432 | 130 |
| Average degree (avgK) | 12.2 | 13.9 | 8.7 |
| Average clustering coefficient (avgCC) | 0.7 | 0.6 | 0.7 |
| Average path distance (GD) | 1.9 | 2.1 | 2.1 |
| Geodesic efficiency (E) | 0.6 | 0.6 | 0.6 |
| Harmonic geodesic distance (HD) | 1.6 | 1.8 | 1.7 |
| Centralization of betweenness (CB) | 0.1 | 0.1 | 0.3 |
| Centralization of stress centrality (CS) | 0.4 | 0.5 | 0.9 |
| Centralization of eigenvector centrality (CE) | 0.5 | 0.6 | 0.5 |
| Maximal closeness centrality | 0.0 | 0.0 | 0.0 |
| Centralization of closeness centrality (CCL) | 0.3 | 0.3 | 0.4 |
| Density (D) | 0.4 | 0.2 | 0.3 |
| Transitivity (Trans) | 0.7 | 0.5 | 0.7 |
| Efficiency | 0.7 | 0.8 | 0.7 |

Table S2 The OTUs linked with OTU_2 in D2 group

| Node | Genus |
| --- | --- |
| OTU_59 | *Pseudogracilibacillus* |
| OTU_13 | *Lactobacillus* |
| OTU_18 | *Bacillus* |
| OTU_258 | *Turicibacter* |
| OTU_30 | *Lactobacillus* |
| OTU_15 | *Thermobifida* |
| OTU_19 | *Lactobacillus* |
| OTU_1668 | *Aeromonas* |
| OTU_33 | *Lactobacillus* |
| OTU_52 | *Streptococcus* |
| OTU_1734 | *Cetobacterium* |
| OTU_21 | *Leuconostoc* |
| OTU_11 | *Leuconostoc* |
| OTU_29 | *Plesiomonas* |
| OTU_12 | *Weissella* |
| OTU_3 | *Vibrio* |
| OTU_1755 | *Weissella* |
| OTU_1213 | *LD29* |
| OTU_38 | *Acinetobacter* |

Table S3 Summary of the RNA sequencing data

| Items | D1 | D2 | D3 |
| --- | --- | --- | --- |
| Total Clean Reads (M) | 42.52±0.65 | 42.97 ±0.52 | 43.14 ±0.23 |
| Total Mapping rate (%) | 92.13±0.12 | 92.08±0.43 | 92.64±0.25 |
| Clean Reads Q20 (%) | 96.06±0.08 | 96.05±0.03 | 96.28±0.07 |
| Clean Reads Q30 (%) | 90.64±0.17 | 90.63±0.08 | 91.03±0.14 |

Table S4 Difference analysis of expressed genes

| Compare Group | Up | Down | Total |
| --- | --- | --- | --- |
| D1-vs-D2 | 30 | 17 | 47 |
| D1-vs-D3 | 1,379 | 672 | 2,051 |
